# Quality assurance of non-coplanar, volumetric-modulated arc therapy employing a C-arm linear accelerator, featuring continuous patient couch rotation

**DOI:** 10.1186/s13014-019-1264-6

**Published:** 2019-04-11

**Authors:** Hideaki Hirashima, Mitsuhiro Nakamura, Yuki Miyabe, Nobutaka Mukumoto, Tomohiro Ono, Hiraku Iramina, Takashi Mizowaki

**Affiliations:** 1 Department of Radiation Oncology and Image-applied Therapy, Graduate School of Medicine, Kyoto University, 54 Kawahara-cho, Shogoin, Sakyo-ku, Kyoto, 606-8507 Japan; 2 Division of Medical Physics, Department of Information Technology and Medical Engineering, Human Health Sciences, Graduate School of Medicine, Kyoto University, 53 Kawahara-cho, Shogoin, Sakyo-ku, Kyoto, 606-8507 Japan

**Keywords:** Non-coplanar VMAT, Continuous couch rotation, Developer mode, Quality assurance

## Abstract

**Purpose:**

To perform quality assurance of non-coplanar, volumetric-modulated arc therapy featuring continuous couch rotation (CCR-VMAT) using a C-arm linear accelerator.

**Methods:**

We planned and delivered CCR-VMAT using the TrueBeam Developer Mode. Treatment plans were created for both a C-shaped phantom and five prostate cancer patients using seven CCR trajectories that lacked collisions; we used RayStation software (ver. 4.7) to this end. Subsequently, verification plans were generated. The mean absolute error (MAE) between the center of an MV-imaged steel ball and the radiation field was calculated using the Winston–Lutz test. The MAEs between planned and actual irradiation values were also calculated from trajectory logs. In addition, correlation coefficients (*r* values) among the MAEs of gantry angle, couch angle, and multi-leaf collimator (MLC) position, and mechanical parameters including gantry speed, couch speed, MLC speed, and beam output, were estimated. The dosimetric accuracies of planned and measured values were also assessed using ArcCHECK.

**Results:**

The MAEs ±2 standard deviations as revealed by the Winston–Lutz test for all trajectories were 0.3 ± 0.3 mm in two dimensions. The MAEs of the gantry, couch, and MLC positions calculated from all trajectory logs were within 0.04°, 0.08°, and 0.02 mm, respectively. Deviations in the couch angle (*r* = 0.98, *p* < 0.05) and MLC position (*r* = 0.86, *p* < 0.05) increased significantly with speed. The MAE of the beam output error was less than 0.01 MU. The mean gamma passing rate ± 2 SD (range) of the 3%/3 mm, 3%/1 mm, and 5%/1 mm was 98.1 ± 1.9% (95.7–99.6%), 87.2 ± 2.8% (80.2–96.7%), and 96.3 ± 2.8% (93.9–99.6%), respectively.

**Conclusions:**

CCR-VMAT delivered via the TrueBeam Developer Mode was associated with high-level geometric and mechanical accuracy, thus affording to high dosimetric accuracy. The CCR-VMAT performance was stable regardless of the trajectory chosen.

**Electronic supplementary material:**

The online version of this article (10.1186/s13014-019-1264-6) contains supplementary material, which is available to authorized users.

## Introduction

Today, 4π radiotherapy is recognized as a useful therapeutic approach ensuring target-dose conformity while sparing doses to organs-at-risk (OARs). The dosimetric advantages of such therapy compared with coplanar intensity-modulated radiotherapy (IMRT) and volumetric-modulated arc therapy (VMAT) have been demonstrated in planning studies for many diseased sites including the brain [1, 2], head and neck [3, 4], liver [5], lung [6], breast [7] and prostate [8]. Recently, 4π radiotherapy including 4π static beam radiotherapy and 4π arc beam radiotherapy has been clinically implemented using mono-isocentric beams. In a Phase 1 trial, Yu et al. showed that 4π static beam radiotherapy was feasible and safe and associated with dosimetric benefits and high-level delivery efficiency when used to treat high-grade glioma [9]. One form of 4π arc beam radiotherapy, O-ring system-specific non-coplanar VMAT, termed Dynamic WaveArc (DWA), has been clinically implemented in the Vero4DRT system (Mitsubishi Heavy Industries, Ltd., Hiroshima, Japan; and BrainLAB AG, Munich, Germany) [10, 11]. DWA is a continuous, non-coplanar beam delivery technique featuring simultaneous gantry and O-ring movement in the absence of couch rotation. High dose conformity and high-level delivery accuracy have been reported by several investigators [12–14].

However, 4π arc beam radiotherapy using a typical C-arm linear accelerator, which has been termed non-coplanar VMAT featuring continuous patient couch rotation (CCR-VMAT) has been investigated in research settings [15–19]. Fahimian et al. and Liang et al. performed trajectory-modulated arc therapy featuring a continuously rotating couch by employing the TrueBeam Developer Mode (Varian Medical Systems, Palo Alto, CA, USA) [15, 16]. While significant dose-sparing of OARs was apparent, the OARs were not considered during optimization, and no gantry rotation occurred during beam delivery [15, 16]. Optimized, continuous, non-coplanar arc trajectory methods have been developed by several research groups using the TrueBeam Developer Mode [17–19]. MacDonald et al. reported that the dose distributions associated with the developed trajectories (calculated using a dedicated treatment planning system [TPS]) did not consider a VMAT scenario featuring simultaneous gantry and couch rotation. In other words, those authors divided the optimized trajectory into sub-arcs and created deliverable plans by smoothing trajectories into multiple non-coplanar sub-arcs without dynamic couch rotation [17]. Wilson et al. developed an in-house software to create arbitrary trajectories and optimized the dose rate and multi-leaf collimator (MLC) leaf sequence [18]. Recently, Fix et al. developed a CCR-VMAT algorithm, including dynamic gantry, couch, collimator rotation and MLC sequence, with continuous movement while the beam is in operation [19]. Final dose distributions were calculated using the Monte Carlo algorithm provided in the commercially available TPS [19, 20]. However, the cited authors focused on the efficiency of VMAT during couch movement and these approaches required a dedicated, specific algorithm in the research environment of TPS. To our best knowledge, few reports have engaged in quality assurance (QA) using several non-coplanar trajectories with couch movement.

Here, we describe a procedure to generate CCR-VMAT plans, including beam setting, optimization and dose calculation, using a commercially available TPS that can create a DWA plan without a specific algorithm. We then implemented the CCR-VMAT plans via the TrueBeam Developer Mode, and assessed the geometric, mechanical and dosimetric accuracies of CCR-VMAT.

## Materials and methods

### CCR-VMAT delivery in the TrueBeam developer mode

**Fig. 1:**
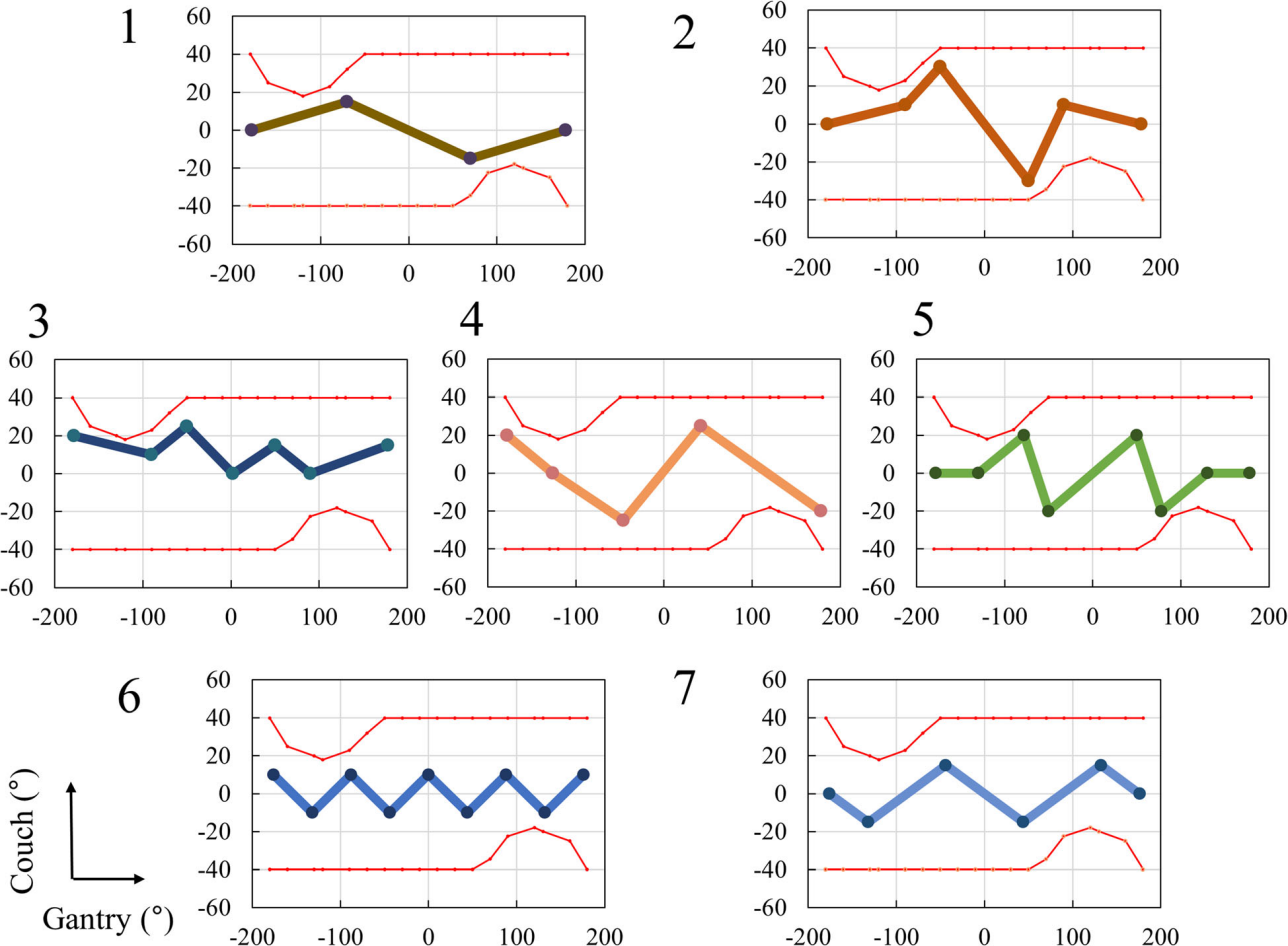
Selected continuous non-coplanar arc trajectories derived using the RayStation treatment planning system. The vertical and horizontal axis show the couch and gantry angles, respectively. The red lines show the collision borders between the gantry head and couch (TrueBeam data)


From 24 trajectories of DWA plans available in RayStation (ver. 4.7; RaySearch Laboratories, Stockholm, Sweden), seven non-coplanar arc trajectories lacking collisions were selected with the help of an in-house collision map prepared for use with TrueBeam when ArcCHECK (Sun Nuclear, Melbourne, FL, USA) was placed on the ExacTrac X-Ray 6D couch (BrainLAB) (Fig. 1). All trajectories featured 4–9 manipulation points, at which the direction of the couch rotation was switched. An additional movie file shows a room-view video of a representative trajectory (trajectory 1; see Additional file 1).

**Additional file 1:** A sample room-view video; CCR-VMAT was operated using a representative trajectory (trajectory 1). (MP4 16236 kb)


To create CCR-VMAT plans for the TrueBeam using RayStation software, we configured the TrueBeam machine model that enables beam delivery with simultaneous rotation of the gantry and couch by changing the geometric properties in the machine constraints workspace of RayStation. No new scripts or codes were required during planning. We then created the CCR-VMAT treatment plans described in the following section. Next, we created eXtensible Markup Language (XML) files for CCR-VMAT irradiation via the TrueBeam Developer Mode. The nominal upper limits of the gantry rotation speed, couch rotation speed, and MLC speed were 6.0°/s, 3.0°/s, and 25 mm/s, respectively. In-house software was used to convert Digital Imaging and Communications in Medicine standard for Radiation Therapy (DICOM-RT) plans to XML files, which were uploaded to the TrueBeam Developer Mode. CCR-VMAT plans were then delivered.

### CCR-VMAT planning based on a C-shaped phantom and prostate cancer data

To confirm the deliverability of CCR-VMAT plans, we created a plan for a C-shaped phantom [21] and for five patients with prostate cancer treated with DWA at our institution. The dose constraints for both plans were described in previous reports [11, 21]. The prescribed doses for the C-shaped phantom and the prostate cancer patients were 50 Gy in 25 fractions and 76 Gy in 38 fractions, respectively. All CCR-VMAT plans were created with dose calculation grid of 2-mm, using a single mono-isocentric arc. Subsequently, verification plans were generated to conduct QA.

### QA of CCR-VMAT

Dosimetric inaccuracies during CCR-VMAT are caused by differences between the mechanical and radiation isocenters as well as mechanical inaccuracy. In this study, we assessed the coincidence of the mechanical and radiation isocenters using the Winston-Lutz test, as well as mechanical accuracy, such as simultaneous MLC, gantry, and couch movement, and beam output, by establishing separate trajectory logs. Thereafter, dosimetric accuracy was verified using ArcCHECK.

### Coincidence of mechanical and radiation isocenters during CCR-VMAT by the Winston–Lutz test

The Winston–Lutz phantom, including a steel ball with a diameter of 3 mm, was placed on the couch in a position defined by an in-room laser. Before the Winston-Lutz test was performed, no collision between the couch and the EPID was visually confirmed for all seven trajectories.

First, two kV images were acquired prior to couch movement using ExacTrac (BrainLAB), to assess the difference between the actual center of the steel ball and the kV-imaged center. Initial setup errors were not corrected. Next, a 6-MV flattened X-ray beam with a field size of 10 × 10 mm^2^ was delivered to obtain electronic portal imaging device (EPID) images in continuous mode while rotating both the gantry and couch, to assess the displacement between the center of the MV-imaged steel ball and the radiation field, as described in detail in the next section. The EPID source-imager distance and pixel size were 1500 mm and 0.22 × 0.22 mm^2^ at the isocenter, respectively. Finally, two more kV images were acquired by ExacTrac after the couch movement, to confirm the reproducibility of the couch position after rotation.

Based on the obtained EPID images, the displacement between the center of the MV-imaged steel ball and the radiation field during CCR-VMAT was calculated using the Winston–Lutz test employing in-house MATLAB R2017b software (MathWorks, Natick, MA, USA). The center of the steel ball was detected using a template-matching method featuring cross-correlation between the MV image and the constructed template. The center of the radiation field was determined as the center of the 50% isodose level on the EPID. The mean absolute errors (MAEs) of the displacement between the center of the MV-imaged steel ball and the radiation field in the cross-plane, in-plane, and two-dimensional directions were calculated for all seven trajectories.

### Mechanical accuracy

The mechanical accuracy of CCR-VMAT was evaluated based on trajectory logs [22]. These logs recorded the commanded and measured positions at 20-ms intervals in terms of gantry angle, couch angle, MLC position, and beam output (the latter in monitor units [MUs]), respectively. The MAEs between the planned and actual trajectory log data were calculated. In addition, correlation coefficients (*r* values) among the MAEs of gantry angle, couch angle, and MLC position, and mechanical parameters including gantry speed, couch speed, MLC speed, and beam output, were estimated.

### Dosimetric accuracy

The calculated and measured dose distributions were assessed by global gamma analysis using ArcCHECK. The criteria were X% of the planned maximum dose as the dose-difference criterion and Y mm as the distance-to-agreement criterion, with a 10% threshold. In this study, global 3%/3 mm, 3%/1 mm and 5%/1 mm gamma were used to assess the dose distribution. The relationships between mechanical errors and passing rates were also evaluated.

## Results

### Coincidence of mechanical and radiation isocenters during CCR-VMAT

**Fig. 2:**
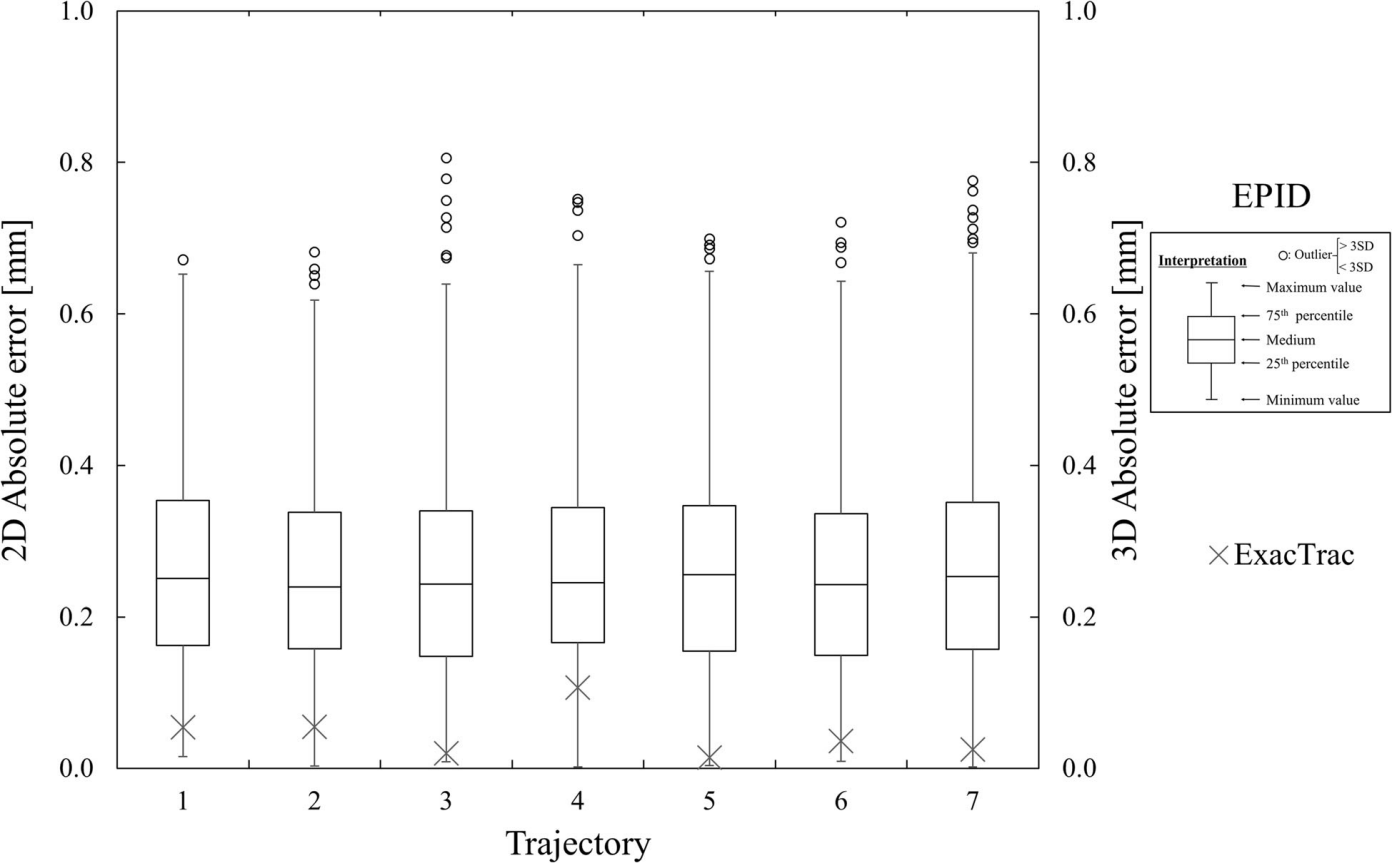
Boxplot of the two-dimensional absolute error by the Winston–Lutz method (measured using the EPID), and three-dimensional absolute error in the central phantom displacement (measured by ExacTrac)

The initial setup errors were less than 0.1 mm for all coordinates. The deviations calculated (employing the Winston–Lutz method) using the laser-to-MV beam, and the central displacement of the kV-imaged phantom for each trajectory. The number of EPID images acquired during CCR-VMAT was 500 to 900 per trajectory. The MAEs ±2 standard deviations (SDs) (thus the maximum deviation) between the center of the MV-imaged steel ball and the radiation field were 0.2 ± 0.2 (0.7) mm, 0.2 ± 0.3 (0.8) mm, and 0.3 ± 0.3 (0.8) mm in the cross-plane, in-plane, and two-dimensional views, respectively. The MAEs ±2 SD (again, the maximum displacement) between the actual center of the steel ball and the kV-imaged center before and after couch movement were 0.01 ± 0.01 (0.02) mm, 0.03 ± 0.05 (0.08) mm, 0.03 ± 0.05 (0.07) mm, and 0.04 ± 0.06 (0.11) mm in the vertical, longitudinal, lateral, and three-dimensional directions, respectively. Figure 2 shows a boxplot of the two-dimensional absolute error calculated by EPID, and the three-dimensional absolute error calculated by ExacTrac.

### Mechanical accuracy

**Fig. 3:**
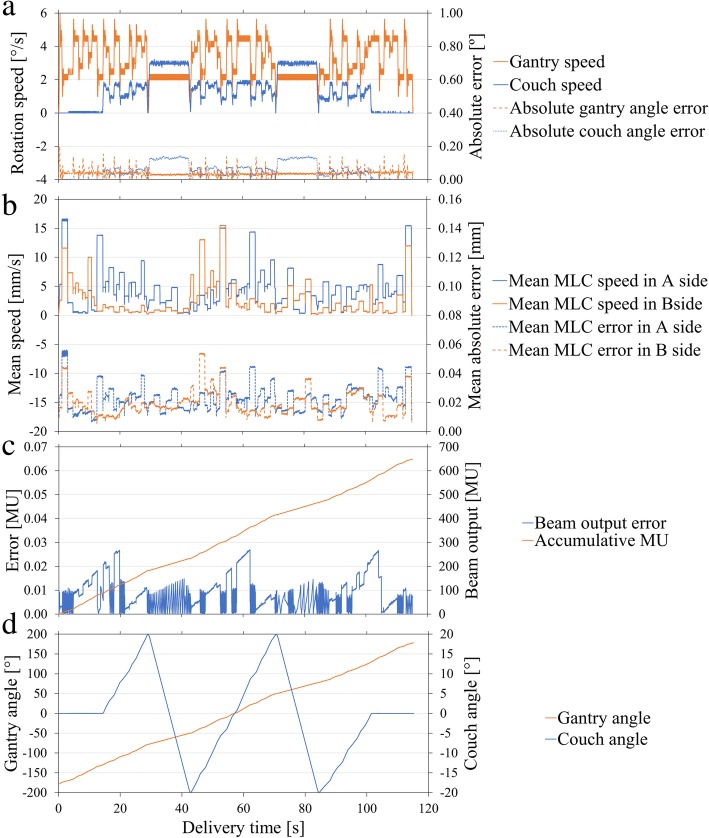
The trajectory log for a representative patient treated using trajectory 5. Mechanical parameters of **a** the gantry and couch, **b** the MLC, **c** the beam output and, **d** the trajectory

**Fig. 4:**
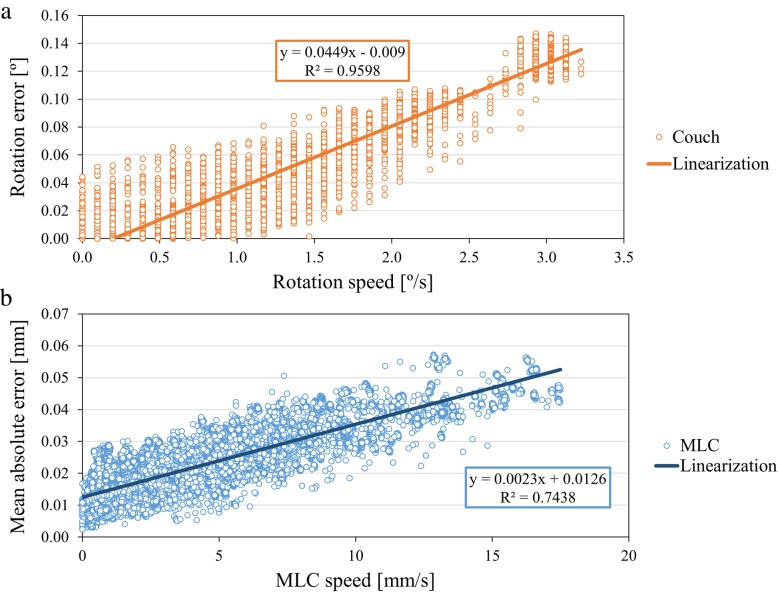
Correlations between mechanical errors and the mechanical speeds of **a** the couch and **b** the MLC. The correlations between couch and MLC errors and speeds were significant (and positive) (*p* < 0.05)

All CCR-VMAT plans met the indicated criteria (Table 1). For all CCR-VMAT plans, the MAEs of mechanical accuracies were within 0.04° for the gantry position, 0.08° for the couch position, 0.02 mm for the MLC position, and 0.01 MU for the beam output. The mechanical accuracies of the gantry angles, couch angles, MLC positions, and beam outputs for all plans are summarized in Table 2. A representative CCR-VMAT trajectory log is shown in Fig. 3. The mean (maximum) speeds of the recorded gantry rotation, couch rotation, and MLC movement derived from the trajectory logs of all trajectories were 1.3 (3.2)°/s, 3.6 (6.0)°/s, and 4.0 (17.5) mm/s, respectively. Figure 4 shows that deviations in couch angle (*r* = 0.98, *p* < 0.05) and MLC position (*r* = 0.86, *p* < 0.05) increased significantly with speed. However, no correlation between the deviation of any mechanical parameter and beam output was apparent.

**Table 1:** Summary of plan quality. Prostate values are shown as the median, with the minimum and maximum values in parentheses

Criteria			Trajectory						
			1	2	3	4	5	6	7
C-shaped phantom									
PTV	D_95%_ (Gy)	≥ 50 Gy (100%)	50.0	50.0	50.1	50.2	50.0	50.0	50.0
PTV	D_max_ (Gy)	≤ 55 Gy (110%)	54.9	54.9	55.0	54.9	55.0	54.9	55.0
Core	D_2cc_ (Gy)	≤ 30 Gy (60%)	29.6	29.7	30.0	29.2	28.9	29.4	28.7
Prostate (*n* = 5)									
PTV	D_mean_ (Gy)	> 75.2 Gy (99%)	76.0 [76.0–76.0]	76.0 [76.0–76.0]	76.0 [76.0–76.0]	76.0 [76.0–76.0]	76.0 [76.0–76.0]	76.0 [76.0–76.0]	76.0 [76.0–76.4]
	D_95%_ (Gy)	> 68.4 Gy (90%)	71.6 [71.2–72.7]	71.5 [71.3–72.7]	71.4 [71.1–72.9]	71.7 [71.6–72.5]	72.5 [71.9–73.1]	71.3 [70.0–72.9]	71.8 [71.1–72.9]
	V_90%_ (%)	> 95%	99.8 [99.2–100.0]	99.8 [99.4–100.0]	99.7 [99.2–99.9]	99.8 [99.6–100.0]	99.9 [99.7–100.0]	99.1 [98.3–99.9]	99.7 [99.2–100.0]
	D_max_ (Gy)	≤ 83.6 Gy (110%)	80.6 [78.7–82.1]	80.9 [79.1–81.6]	81.0 [80.4–81.8]	80.7 [80.4–81.5]	81.1 [79.5–83.0]	81.5 [79.8–81.8]	81.0 [80.6–83.6]
Bladder wall	V_40Gy_ (%)	< 65%	22.0 [11.8–31.4]	22.9 [11.8–28.8]	21.2 [13.3–32.5]	22.0 [11.5–27.7]	24.9 [11.8–29.6]	26.0 [13.2–30.5]	25.3 [13.7–32.0]
	V_70Gy_ (%)	< 35%	12.1 [5.5–17.0]	12.1 [5.6–17.5]	11.3 [5.6–17.0]	12.1 [5.5–16.7]	12.3 [5.7–17.0]	12.2 [6.1–16.9]	12.2 [6.0–16.6]
Rectal wall	V_40Gy_ (%)	< 65%	40.3 [27.6–41.0]	40.4 [28.5–42.0]	40.3 [27.6–43.9]	39.3 [29.2–41.6]	37.0 [29.9–42.7]	40.7 [28.1–43.3]	40.9 [28.8–43.1]
	V_60Gy_ (%)	< 35%	24.8 [12.5–26.0]	23.8 [12.1–26.8]	23.7 [12.5–26.7]	23.6 [13.7–25.8]	21.2 [13.1–24.8]	24.7 [12.3–25.5]	23.6 [12.9–25.9]
	V_70Gy_ (%)	< 25%	9.2 [3.7–13.6]	9.5 [3.4–14.0]	9.6 [2.7–14.2]	9.2 [4.0–11.8]	11.6 [4.6–11.7]	9.1 [3.3–12.1]	11.4 [3.7–12.4]
	V_76Gy_ (%)	< 1%	0.0 [0.0–0.0]	0.0 [0.0–0.0]	0.0 [0.0–0.0]	0.0 [0.0–0.0]	0.0 [0.0–0.0]	0.0 [0.0–0.0]	0.0 [0.0–0.0]

Abbreviations: *D*_*xx%(cc)*_ dose covering xx% (cc) of the volume, *D*_*max*_ maximum dose, *V*_*yy Gy*_ volume receiving yy Gy, *V*_*yy%*_ volume receiving yy % isodose, *PTV* planning target volume

**Table 2:** The mechanical accuracies of gantry angle, couch angle, MLC position, and MU. The mean absolute errors ±2 standard deviations for the C-shaped phantom and the prostate cancers are shown

Trajectory	C-shaped phantom				Prostate cancers (*n* = 5)			
	Gantry [°]	Couch [°]	MLC [mm]	Beam output [MU]	Gantry [°]	Couch [°]	MLC [mm]	Beam output [MU]
1	0.04 ± 0.01	0.01 ± 0.01	0.03 ± 0.04	0.01 ± 0.02	0.04 ± 0.03	0.04 ± 0.02	0.02 ± 0.03	0.01 ± 0.01
2	0.04 ± 0.02	0.03 ± 0.06	0.03 ± 0.05	0.01 ± 0.02	0.04 ± 0.04	0.05 ± 0.08	0.02 ± 0.03	0.01 ± 0.02
3	0.04 ± 0.02	0.04 ± 0.09	0.03 ± 0.05	0.01 ± 0.01	0.04 ± 0.02	0.03 ± 0.06	0.02 ± 0.03	0.01 ± 0.01
4	0.04 ± 0.01	0.03 ± 0.03	0.03 ± 0.04	0.01 ± 0.02	0.04 ± 0.03	0.07 ± 0.05	0.02 ± 0.04	0.01 ± 0.01
5	0.04 ± 0.02	0.04 ± 0.08	0.03 ± 0.05	0.01 ± 0.01	0.04 ± 0.04	0.06 ± 0.09	0.02 ± 0.03	0.01 ± 0.01
6	0.04 ± 0.01	0.02 ± 0.01	0.02 ± 0.03	0.01 ± 0.02	0.04 ± 0.03	0.08 ± 0.03	0.02 ± 0.04	0.01 ± 0.01
7	0.04 ± 0.02	0.04 ± 0.01	0.03 ± 0.04	0.01 ± 0.02	0.04 ± 0.03	0.06 ± 0.02	0.02 ± 0.04	0.01 ± 0.01

Abbreviations: *CCR-VMAT* non-coplanar, volumetric-modulated arc therapy featuring continuous couch rotation, *MLC* multi-leaf collimator, *MU* monitor units

### Dosimetric accuracy

**Fig. 5:**
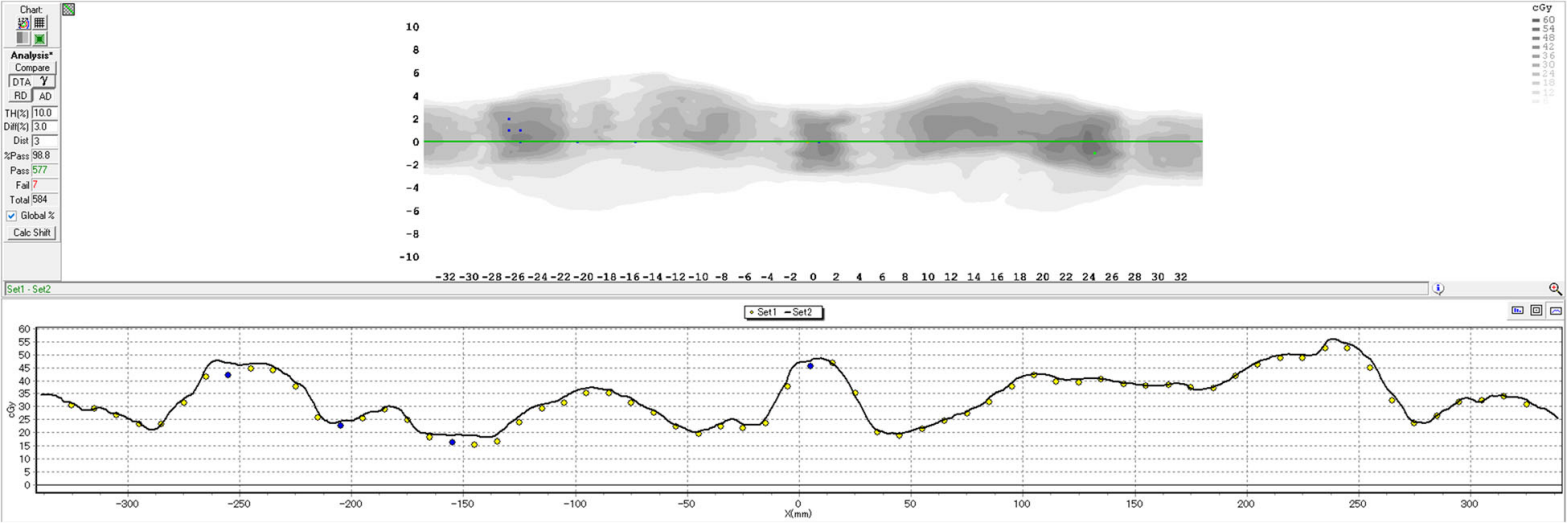
An example of a gamma analysis on an ArcCHECK phantom using trajectory 1. The gamma map and dose difference profiles are shown

The gamma passing rates of the CCR-VMAT plans for the C-shaped phantom and prostate cancers are shown in Table 3. In all cases, the mean gamma passing rate ± 2 SD (range) of the 3%/3 mm, 3%/1 mm, and 5%/1 mm was 98.1 ± 1.9% (95.7–99.6%), 87.2 ± 2.8% (80.2–96.7%), and 96.3 ± 2.8% (93.9–99.6%), respectively. Figure 5 shows the gamma maps and dose difference profiles of the representative prostate case with trajectory 1. We found no correlation between the MAE of any mechanical parameter and any gamma passing rate.

**Table 3:** Gamma passing rate of CCR-VMAT plans for the C-shaped phantom and prostate cancers. The prostate cancer data are means ±2 standard deviations

Trajectory	C-shaped phantom			Prostate cancers (n = 5)		
	3%/3 mm [%]	3%/1 mm [%]	5%/1 mm [%]	3%/3 mm [%]	3%/1 mm [%]	5%/1 mm [%]
1	98.0	91.5	97.5	98.3 ± 1.7	86.5 ± 2.8	95.9 ± 1.4
2	99.1	92.1	98.6	98.2 ± 0.5	84.9 ± 0.5	95.9 ± 2.2
3	99.2	92.0	98.5	98.4 ± 1.9	85.8 ± 1.8	95.8 ± 0.9
4	99.6	96.7	99.6	98.5 ± 0.8	88.2 ± 4.8	96.5 ± 2.8
5	99.0	92.5	98.3	97.8 ± 1.0	86.0 ± 3.8	96.0 ± 2.5
6	98.9	90.2	98.1	97.5 ± 1.6	85.8 ± 3.6	95.9 ± 2.4
7	99.5	95.8	99.2	97.2 ± 2.3	87.1 ± 5.7	95.8 ± 1.8

Abbreviation: *CCR-VMAT* non-coplanar, volumetric-modulated arc therapy featuring continuous couch rotation

## Discussion

We generated CCR-VMAT plans without using a specific algorithm and performed QA for seven continuous, non-coplanar arc trajectories that lacked collisions, using the TrueBeam Developer Mode. The mechanical errors including those of gantry angle, couch angle, MLC position, and beam output during CCR-VMAT were comparable with those of previous reports [10, 18, 22, 23]; the dosimetric accuracies met the criteria of the American Association of Physicists in Medicine Task Group 119 [24].

Wilson et al. used an EPID to reveal the geometric inaccuracies caused by CCR. They determined that the Winston–Lutz differences between the static and couch rotation conditions were 0.2 ± 0.1 mm and − 0.1 ± 0.3 mm in the cross- and in-plane directions, respectively [22]. The laser-to-MV beam errors in synchronized gantry-couch rotation were 0.2 ± 0.2 mm and 0.2 ± 0.3 mm in the cross- and in-plane directions, respectively. Our experiments were more complicated than those of an earlier study since the direction of couch rotation switched 4–9 times during gantry rotation [22]; however, the deviations were comparable to those noted in that report and were stable even when continuous non-coplanar trajectories were employed. Moreover, the displacement of the laser-to-kV isocenter measured by ExacTrac before and after couch movement was negligible (less than 0.1 mm); therefore, couch position was highly reproducible.

Wilson et al. also reported that couch angular positions in dynamic couch rotation recorded in trajectory logs were accurate to within 0.05° [22]. Moreover, the root mean square errors of gantry and couch positions in simultaneous gantry-couch rotation attained 0.05° and 0.06°, respectively [18]. Burghelea et al. found that the mean deviations of gantry and ring angles in DWA were − 0.03° ± 0.46° and 0.18° ± 0.26°, respectively [10]. These results were comparable with the deviations of gantry and couch positions in CCR-VMAT recorded in our trajectory logs. Even when the MLC positions and beam outputs varied by the trajectories, the mechanical accuracy was comparable with that found in previous reports [23]. We found a high correlation between mechanical error and speed, indicating that restricting the mean or maximum mechanical speeds reduced mechanical errors, as reported previously [23]. In addition, we found no correlation between the deviation of any mechanical parameter and beam output, suggesting that the output was well controlled (thus not affected by any mechanical parameter). In terms of verification using a trajectory log, Neal et al. reported a clinically evident discrepancy between the image-based and trajectory log-based MLC positions [25]. We carefully verified this before commencing our study. We included an intentional error, which confirmed that the difference between the EPID measurement and the trajectory log was within the allowable range. Therefore, trajectory-log verification was deemed to be of adequately accurate.

In general, continuous, non-coplanar arc plans that optimize conformal dose distributions are not easily formulated, because it is difficult to avoid collisions between the gantry and couch. Existing, applicable, clinical non-coplanar arc plans are time-consuming because of the need to rotate the patient couch manually. It is well established that CCR-VMAT provides dose distributions, thus enabling the concentrated administration of a sufficient dose within the target volume while minimizing the dose in the surrounding OARs, in addition to the benefit of time-saving [3, 7, 15–19]. For safe delivery of CCR-VMAT plans, dosimetric verification should be a requirement. The average passing rates at the 3%/3-mm gamma criterion, applicable in continuous clinical non-coplanar arc delivery, were greater than 97% for several trajectories [10]. Our experimental results were comparable with the clinical results, despite the combination of TPSs and treatment machines from different vendors [26]. Therefore, our approach can be expected to provide clinically acceptable and deliverable CCR-VMAT plans.

## Conclusion

CCR-VMAT delivered via the TrueBeam Developer Mode was associated with high-level geometric and mechanical accuracy, which in turn afforded high dosimetric accuracy. The CCR-VMAT performance was stable regardless of the trajectory chosen.

## Additional files

Additional file 2: The dataset supporting our findings. (XLSX 3027 kb)

## Abbreviations

CCR-VMAT: Non-coplanar VMAT featuring continuous couch rotation
DICOM-RT: Digital Imaging and Communications in Medicine standard for Radiation Therapy
DWA: Dynamic WaveArc
EPID: Electronic portal imaging device
IMRT: Intensity-modulated radiotherapy
MAEs: Mean absolute errors
MLC: Multi-leaf collimator
MUs: Monitor units
OARs: Organs at risk
PTV: Planning target volume
SD: Standard deviation
TPS: Treatment planning system
VMAT: Volumetric modulated radiation therapy
XML: eXtensible Markup Language
